# Comparison of child self-reports and parent proxy-reports on quality of life of children with attention deficit hyperactivity disorder

**DOI:** 10.1186/1477-7525-11-186

**Published:** 2013-11-04

**Authors:** Juliana CB Marques, Jorge A Oliveira, Juliana B Goulardins, Roseane O Nascimento, Allana MV Lima, Erasmo B Casella

**Affiliations:** 1 Departament of Pediatrics, Child Institute, Faculty of Medicine of University of São Paulo, Av. Dr. Enéas Carvalho de Aguiar, 647, São Paulo, SP CEP: 05403.000 Brasil; 2 Motor Behavior Laboratory, School of Physical Education and Sports, University of São Paulo, Av. Prof. Mello Moraes, 65, Cidade Universitária, SP CEP: 05508-030 São Paulo, Brazil

**Keywords:** Quality of life, Attention deficit hyperactivity disorder, Child, Self-concept

## Abstract

**Background:**

Attention deficit hyperactivity disorder (ADHD) is a neurobiological condition that affects 3%–7% of the pediatric population and significantly compromises the quality of life (QoL) of these individuals. The aim of the current study was to compare child self-reports and parent proxy reports on the QoL of children with ADHD.

**Methods:**

Forty-five children with ADHD, combined type, aged 8–12 years without comorbidities, were compared with 43 typically developing children. PedsQL™ 4.0 (Pediatric QoL Inventory™) Generic Core Scales (physical, emotional, social, and school functioning) were completed by families and children self-reporting their health-related QoL.

**Results:**

Children with ADHD reported themselves significantly lowered their PedsQL™ scores on all dimensions in comparison to typically developing children. Statistically significant differences were observed in social functioning (p = 0.010), school functioning (p <0.001), psychosocial health (p <0.001), and total score (p = 0.002). The physical functioning and emotional functioning dimensions did not differ significantly between groups, with p = 0.841 and p = 0.070, respectively. Parents of children with ADHD also reported lower PedsQL™ scores, with statistically significant differences in all dimensions. The relationship between child self-reports and parent proxy reports indicated that there is greater agreement among children with ADHD, except for the school functioning.

**Conclusions:**

This suggests that children with the disorder and their parents have a perception of the functional limitations the disorder brings. It is therefore important to undertake studies to verify the QoL in children with ADHD that aim to provide and measure the scope of the well-being of these children.

## Background

Attention deficit hyperactivity disorder (ADHD) is the most common neuropsychiatric disorder during childhood [1], with the worldwide-pooled prevalence estimated to be 5.29% [2]. Although the prevalence estimates reported in individual studies have varied widely, the pooled results suggest that the prevalence of ADHD is similar (5.9%–7.1%), whether ADHD is defined by parent ratings, teacher ratings, or a best-estimate diagnostic procedure in children and adolescents [3].

The disorder is currently a common reason for the referral of children to medical and mental health professionals, representing approximately 30%–50% thereof. For this reason, it is considered one of the most serious public health problems [4,5], and it causes great economic harm to the children and families dealing with this disorder, as well as to society [6].

The disorder tends to have a chronic course and is associated with a number of complications in childhood, adolescence, and adulthood. It has been highlighted that 50%–70% of these children mature into symptomatic adults [7,8].

ADHD individuals suffer not only from symptoms of inattention, hyperactivity, and impulsivity, but often show losses in various contexts, including motor skills, academic performance, school behavior, peer relations, and family functioning. This indicates a great loss in many parameters of quality of life (QoL), mainly due to psychosocial health, directly influencing the well-being of their subjects. Usually these difficulties persist into adulthood, damaging the daily routine and decreasing self-esteem [9-12].

According to the World Health Organization, QoL is defined as “an individual’s perception of their position in life in the context of the culture and value systems in which they live and in relation to their goals, expectations, standards and concerns” [13]. The assessment of QoL in adults is now well established and these measures are routinely included in many clinical trials [14], but to date, a surprisingly limited number of studies have utilized QoL measures to investigate the specific effects on adults with ADHD, although there appears to be a consensus that adults with ADHD have a lower QoL than non-ADHD individuals [15].

The possibility of acquiring knowledge in these areas in children with different health situations allows us to identify important changes in the epidemiology of childhood illness [14]. Although ADHD seriously compromises QoL, few studies have provided specific tests to measure QoL in children with ADHD [16]. This fact can be verified by the lower use of instruments for QoL in children with mental disorders than in children with physical disabilities [17]. There is also little information in the research literature as to the extent of the agreement between parent and child in the reports of child QoL for a sample of children diagnosed with ADHD [18].

Understanding the impact of mental disorders, particularly ADHD, on QoL, allows us to observe different levels of impairment. This kind of thinking is reinforced by the demonstration that mental health problems often inflict greater damage on QoL than physical disorders [19,20].

Knowledge about the domains of QoL that affect children with psychiatric disorders may provide a diagnosis and enable appropriate treatment goals to be set [21], while promoting educational and psychosocial development for children with ADHD [13]. Hence, there is a need for studies to verify QoL in children with ADHD that can generate more appropriate means of intervention aimed at measuring and enhancing the scope of the well-being in these children [11,22].

Health-related QoL (HRQoL) is a multidimensional measure of the overall condition of a human life, both physical and psychosocial [13,23]. The HRQoL comprises disease- and treatment-related aspects of the individual, such as pain, limitations in motor ability, energy level, or mood [15]. HRQoL is an important outcome that has received little attention in children with ADHD [18] because the effect of this disorder on the daily routine and well-being remains underexplored. For a better understanding of these factors, it is necessary to use instruments to assess at least the physical, social, and emotional domains, as outlined by the World Health Organization [13].

Research studies about QoL in ADHD have demonstrated that the agreement between the child and parent ratings for physical health is greater than that for psychosocial health [14,24]. Due to the small number of studies, and given the discrepancies between parent and child reports, measuring the perspectives of both parent and child seems appropriate. The aim of the current study was to compare child self-reports and parent proxy reports of QoL of children with ADHD.

## Materials and methods

### Subjects

A cross-sectional study was conducted on 88 children, aged 8 to 12 years, who were separated into two groups: the ADHD group and a control group.

The ADHD group comprised 45 treatment-naïve patients with clinical diagnoses of ADHD, combined type, according to classification criteria in the DSM-IV-TR, and without psychiatric comorbidities, except oppositional defiant disorder (ODD) [1]. Comorbidity with ODD was not an exclusion criterion for this study due its high frequency in children with ADHD. These inclusion criteria were chosen in order to maintain the homogeneity of the samples. The children were treated by a multidisciplinary team and follow-up treatment was provided at the Learning Disorders Clinic of the Institute for Children, Hospital of the Faculty of Medicine, University of Sao Paulo (HCFMUSP), Sao Paulo, Brazil.

The control group had 43 typically developing children, aged 8–12 years, from public schools. This group was similar in terms of gender, family income, family income, parents’ marital status, and parental education.

The exclusion criteria for both groups were mental retardation; visual, hearing, heart, rheumatic, orthopedic, neurological, or severe behavioral disorders; and regular use of medications.

Written consent was obtained from the parents and/or caregivers of all participants, and the study was approved by the Ethics Committee of the University of São Paulo Faculty of Medicine (n. 0572/08).

### Measures

#### *Identification form prepared by the researchers*

This form included family income of below minimum wage, between one and three times minimum wage, between four and 10 times minimum wage or above 10 times minimum wage; degree of parental education divided into never having studied or studied for four years, elementary school, high school, university, or postgraduate students; and marital status of parents as either single, married, or divorced.

#### *SNAP-IV questionnaire*

This is a public domain tool developed to assess the symptoms of ADHD in children and adolescents. It uses the 18 symptoms listed in the DSM-IV-TR [25,26].

#### *Pediatric QoL inventory™ (PedsQL™ 4.0) generic core scales*

This is a brief, standardized assessment instrument that systematically assesses HRQoL through child self-reports and parent proxy report scales. The generic core scales were chosen for this study due to their translation, adaptation, and validation in Brazil [27,28]. They consist of 23 items applicable to a healthy school and community populations, as well as pediatric populations with acute and chronic health conditions. The 23-item PedsQL™ 4.0 generic core scales encompass: 1) physical functioning (eight items); 2) emotional functioning (five items); 3) social functioning (five items); and 4) school functioning (five items). The instrument takes approximately five minutes to complete. The items for each form are essentially identical, differing in developmentally appropriate language, or first- or third-person tense. The instructions ask how much of a problem each item has been during the past month. A 5-point response scale is utilized across the child self-reports for ages 8–18 and for the parent proxy report (0 = never a problem; 1 = almost never a problem; 2 = sometimes a problem; 3 = often a problem; and 4 = almost always a problem). Items are reverse scored and linearly transformed into a 0–100 scale (0 = 100, 1 = 75, 2 = 50, 3 = 25, and 4 = 0) so that higher scores indicate better HRoL [27,28].

### Procedures

Children with ADHD were identified via an initial screening process in which their teachers and parents were asked to complete the SNAP-IV questionnaire. Then an expert pediatric neurologist submitted these children to further assessment using DSM-IV-TR criteria [1]. From these diagnoses, ADHD types were identified. Once we confirmed the ADHD diagnosis, ADHD type, and absence of comorbidities (except ODD), all patients were assessed by the main researcher at the Learning Disorders Clinic. The identification form and the PedsQL™ parent proxy report was answered by the parents and/or caregivers individually through interviews. Children were interviewed separately with PedsQL™ for child self-reports.

In order to ensure that the children selected for the control group were free of symptoms of hyperactivity, impulsiveness, or inattention, we based our inclusion criteria on the information provided by their parents in the SNAP-IV questionnaire. The children were assessed with PedsQL™ child self-reports at school, during the class, for about 10 minutes in a room isolated from other colleagues. Parents were contacted by telephone by the main researcher to complete the identification form and the PedsQL™ parent proxy reports.

### Statistical analysis

Initially, the Shapiro-Wilks test was used to determine the framework of numerical variables in a model with a Gaussian distribution. Given the results presented in this test, it was found that not all variables had numerical parametric distributions. Thus, mean values and confidence intervals of 95% (95% CI) were presented as descriptive statistics. Subsequently, the Student *t*-test for independent data and U Mann–Whitney comparisons established between the mean values, respectively, were observed for both sexes. We used the Bland-Altman method in order to verify the agreement between the responses of the children and their parents for each domain of QoL. The Bland-Altman method calculates the mean difference between two methods of measurement, with 95% limits of agreement as the mean difference. It is expected that the 95% limits include 95% of differences between the two measurement methods.

For the analyses between groups with respect to categorical variables, we used the chi-square test for the linear trend (ordinal variables) and heterogeneity (nominal variables). The criterion for statistical significance was set at 5%. Stata version 11.0 (Stata Corporation, College Station, TX, USA) was used for all statistical calculations.

## Results

### Comparison between groups

The criteria for comparisons between groups were age, gender, family income, parents’ marital status, and father’s education, and mother’s education. There was no statistical difference between the groups in terms of gender, family income, parents’ marital status, father’s education, or mother’s education. However, a statistically significant difference was identified in the variable age (p = 0.038), as shown in Table 1.

**Table 1:** Comparisons between groups

	**Mean age**	**CI 95%**	***p***
ADHD group	9.79	9.35 – 10.22	0.038*
Control group	9.16	8.80 – 9.51	
Gender	Female	Male	*p*
ADHD group	10	35	0.674*
Control group	8	35	
Family Income	≤ 03 SM (%)	> 04 SM (%)	*p*
ADHD group	53.66	46.34	0.679*
Control group	58.14	41.86	
Marital status	Married (%)	Single/Divorced (%)	*p*
ADHD group	53.66	46.34	0.069*
Control group	72.09	27.91	
Father’s education	ADHD group (%)	Control group (%)	*p*
> 08 years	56.10	72.09	0.250*
05 - 08 years	14.63	4.65	
≤ 04 years	29.27	23.26	
Mother’s education	ADHD group (%)	Control group (%)	*p*
> 08 years	73.17	72.09	0.879*
05 - 08 years	9.76	9.30	
≤ 04 years	17.07	18.60	

*Chi Square Test.

### Comparison of the results obtained in evaluating QoL

Among the children’s self-reports, the group with ADHD had lower scores than the control group in all dimensions assessed. Statistically significant differences were observed in social functioning, school functioning, psychosocial health, and total score (Table 2).

**Table 2:** Comparisons of children’s self-reports of QolL - PedsQL™

	**ADHD group**			**Control group**			
**Domains**	**Mean**	**SD**	**CI 95%**	**Average**	**SD**	**CI 95%**	**p**
Physical functioning	78.56	2.41	73.69 - 83.43	80.88	1.59	77.67 - 84.09	0.841*
Emotional functioning	63.33	3.04	57.18 - 69.47	70.66	2.56	65.48 - 75.85	0.070**
Social functioning	65.77	3.97	57.76 - 73.79	81.16	1.82	77.48 - 84.83	0.010*
School functioning	63.22	2.76	57.64 - 68.79	83.95	1.88	80.14 - 87.76	< 0.001*
Psychosocial health	64.10	2.73	58.60 - 69.61	78.42	1.59	75.21 - 81.64	< 0.001*
Total score	69.15	2.37	64.37 - 73.92	79.28	1.42	76.39 - 82.16	0.002*

*Mann–Whitney Test, **T Student’s Test.

In the analysis of the reports of the parents, the group with ADHD had lower scores in all dimensions. This difference was significant in all areas, as can be seen in Table 3.

**Table 3:** Comparison of parents proxy-reports of QoL - PedsQL™

	**ADHD group**			**Control group**			
**Domains**	**Mean**	**SD**	**CI 95%**	**Mean**	**SD**	**CI 95%**	**p**
Physical functioning	79.32	2.99	73.29 - 85.35	92.56	1.62	89.28 - 95.83	< 0.001*
Emotional functioning	62.77	2.35	58.02 - 67.53	76.97	2.61	71.70 - 82.24	< 0.001**
Social functioning	69.92	3.90	62.05 - 77.80	93.72	1.47	90.73 - 96.70	< 0.001*
School functioning	48.66	2.82	42.97 - 54.35	88.83	1.98	84.82 - 92.85	< 0.001*
Psychosocial health	60.45	2.21	55.98 - 64.91	86.42	1.40	83.58 - 89.27	< 0.001*
Total score	67.10	2.21	62.63 - 71.57	88.64	1.29	86.02 - 91.26	< 0.001*

*Mann–Whitney Test, **T Student’s Test.

### Analysis of correlation between parents and children in the ADHD and control groups

The agreement between parents and children was analyzed using the Bland-Altman test. In this analysis, we found the group with ADHD showed greater agreement between parents and children than the control group, except in school functioning, in which children with ADHD had higher scores on mean of 14.55, according to the parents, suggesting that the perception of difficulty in this area is different for the group of children with ADHD (Table 4).

**Table 4:** Agreement between parents and children analyzed using the bland-Altman test

	**ADHD group**			**Control group**		
**Domains**	**Mean**	**CI 95%**	**SD**	**Mean**	**CI 95%**	**SD**
Physical functioning	-0.76	-6.44 4.92	2.82	-11.67	-16.16 -7.18	2.22
Emotional functioning	0.55	-5.96 7.07	3.23	-6.30	-13.45 0.83	3.54
Social functioning	-4.14	-12.51 4.21	4.14	-12.55	-16.78 -8.33	2.09
School functioning	14.55	7.77 21.33	3.36	-4.88	-10.22 0.46	2.64
Psychosocial health	3.65	-1.43 8.74	2.52	-8.00	-11.90 -4.09	1.93
Total score	2.04	-2.49 6.58	2.25	-9.36	-12.93 -5.79	1.76

## Discussion

This study assessed the HRQoL of children with ADHD according to the children’s self-reports and the perceptions of their parents, compared with typically developing children. We also analyzed the relationship agreement between the results of the children’s self-reports and parental reports to determine if there is good awareness of the impact of the disorder on QoL in these children compared with the control group.

We observed that between groups, the only variable with a statistically significant difference was age, which reflects a limitation of this study. However, we believe that this is not a factor of great impact on the results because the mean age was around nine years. Another factor that reinforces the hypothesis of the low impact of this difference in results is that the ADHD group was composed of children slightly older than the control group. If we consider the results of Goulardins et al. [10] that reveled an immaturity in motor development of children with ADHD (negative age -12.8 months), and Shaw et al.’s study [29] that showed that ADHD is characterized by the delay in cortical maturation, then we can consider that the groups have equivalent skills and maturity.

In agreement with other studies [12,22,24,30], our results indicated that children with ADHD have lower QoL, with the condition directly interfering in their well-being, socialization, and self-esteem, in addition to academic skills and learning abilities.

Analyzing each domain separately, we found that children with ADHD, according to their self-reports, have no perception of impairment in their physical functioning. However, when comparing parents’ proxy reports (ADHD group vs. the control group), there were significant differences, which is close to the result from the children’s self-reports. These results suggest that children with the disorder do not, according to their perception, experience prejudice or difficulty in physical functioning, showing good agreement with their parents, with a minimal average difference between the scores. This is not so with the control group, in which there was a great variation between the average scores of children and their parents and/or caregivers, suggesting that, according to the parents of these children, they have superior physical functioning over what the children evidence in their self-evaluation.

The results concerning the correlation between children with ADHD and their parents indicate a good perception of the disorder’s impact on the issues assessed, since the difference between the scores was smaller in the study group. In this regard, they resemble the results of Klassen, Miller, and Fine [24], who assessed the QoL of 58 children diagnosed with ADHD and parent proxy reports. These authors made use of a different assessment tool: the Child Health Questionnaire (CHQ). This questionnaire also has a field related to physical capacity. This study suggested that for observational issues — the term used by the authors to determine physical issues — the agreement between the groups was greater than for non-observational issues (self-esteem, well-being, mental health, and behavior).

Significant differences in the emotional functioning field were only found in the parents’ proxy reports, while the children’s self-reports scored lower in the ADHD group, but showed no statistically significant difference. It suggested that children with ADHD have significant losses on issues related to their feelings and desires, especially from the view of their parents. These children show greater difficulty regulating these areas, presenting a significant risk for other neuropsychiatric disorders, such as depression and anxiety [24,30-33].

In our study, both children with the disorder and their parents have a sense of its emotional impact in their lives, since the agreement between the ADHD groups had the best among all domains and scores analyzed. This did not occur with the control group.

The scientific literature about the social functioning of these children has already contained several studies [22,24,27,34] indicating an important impairment in this dimension. This suggests that the expression of ADHD symptoms could result in “social disability” as expressed by a downward shift in social skills that would otherwise not be expected in children, adolescents, and adults based on scores of normal intelligence.

It has been widely documented that children with symptoms of ADHD are more likely to show social impairment in several areas, including peer relationships, relationships between siblings, and parent–child relationships. Weiss and Hechtman [35] reported that young people with ADHD tended to have fewer friends and experience intense difficulty in having a relationship with the opposite sex, leading to a poor QoL that will clearly jeopardize their future career, family, social, and academic prospects, as well as all other interfaces in the life of any individual [9,22,36-38].

The ADHD group showed a statistically significant difference in social functioning in their self-assessment compared with the group without the disorder, with, demonstrating the fragility the ADHD group shows in this area. The results are similar to the parents’ reports, showing a highly significant difference. Thus, our results are in agreement with the international literature, such as the study by Topolski et al. [39] that identified similar values by evaluating the instrument generic Quality of Life Youth Instrument-Research Version (YQOL R) on adolescents with ADHD compared with a control group of teens and a group of adolescents with mobility limitations. They determined that the group with ADHD had deficits in the dimensions of self-domain and relationships when compared with other groups. As a consequence of these findings we started to look as a whole to the child and the adolescent with the disorder, serving to comprehend these patients not only as one limitation, but rather broad issues that should have appropriated intervention.

The concordance between the children’s self-reports and parental reports in the ADHD group was also adequate, while in the control group they diverged more. Thus, we consider there was good communication within the group with the disorder, as evidenced by parents’ awareness of the difficulties of relationships between their children and colleagues and accompanying activities with their peers. For this reason, they sought medical help.

For the evaluation of the Scholar function, we can see the huge impact that this dimension has on the lives of these children and the effects it can generate in other domains. Our results demonstrated no significant difference between the children’s self-reports and those of their reporting parents. All of this is consistent with the literature, pointing to a correlation between ADHD and poor academic performance [22,30].

This was the only issue in which the control group showed a better correlation than the ADHD group. In this case, the ADHD group had a high mean difference, indicating they believe to have better school performance compared with their parents’ reports. We believe, though, the children realize their difficulty, because there was a significant difference between the comparison with control group. However, they do not consider their academic problems as important as their parents’ perceptions. Possibly the question of the immaturity of the children interferes with these values, and we cannot ignore the perception of parents towards these difficulties can also be exacerbated.

The PedsQL™ 4.0 Generic core scales show two global scores: psychosocial health (encompassing emotional, social, and school) and total score (physical, emotional, social, and school). Comparing the child self-reports among the ADHD group and the control group, it was noticed a significant difference. The psychosocial health is the most impaired score. Parents’ proxy reports showed an exacerbated difference. The parents of the control group see a much higher QoL of their children, differently from the parents of ADHD group as well as the children’s perception.

Another limitation of this study was the inability to exclude children with ODD, showing how high this disorder coexists with ADHD, approximately 60% [9,40]. In addition, there were difficulties to pair children coming from private and public institutions since the control group was only collected at public schools, as the socio-economic context is very different.

Further studies should be conducted for assessing issues related to QoL, well-being, self-esteem, and psychosocial functioning, among others, in addition to investing in interventions that may act directly on these issues, which are crucial for social, family, and emotional well-being.

We identify and emphasize the importance of this issue in the daily lives of children with ADHD reflecting in their adolescence and adulthood, so that intervention strategies must be devised to have a positive impact on the QoL of these people and prevent greater losses. As demonstrated by this and several other studies, the negative impact that this condition can generate in theirs and their families’ lives is profoundly real.

This study revealed the high correlation between parents and children and demonstrated that individuals with ADHD have a good understanding of their condition and their parents are aware of the needs of their children. The comprehension that the children with ADHD can self-evaluate will assist health professionals to change the child’s role in the therapeutic and diagnoses process from passive to an active being with full understanding of their limitations and possibilities.

The ADHD group showed impairments in all QoL domains in the children’s self-reports, with significant differences compared with the controls as being evident in the following areas: social functioning, school functioning, psychosocial health, and total score.

The parental reports revealed that, according to their perceptions, there is a difference in all aspects assessed, including in the domains physical functioning and emotional functioning.

The correlation between the children’s self-reports and the parents’ reports indicated that there is more agreement within the group with ADHD, except in school functioning. This data indicates that a child with the disorder and the parents have a perception of the functional limitations caused by this disease.

## Competing interests

The authors declare that they have no competing interests and this study had no financial support.

## Authors’ contributions

CBM and JBG carried out the initial screening process, collected data and draft the manuscript. EBC evaluated the children and did the medical diagnosis. RON, AMVL and JAO did the statistical analyses and drafted the manuscript. All authors read and approved the final manuscript.
